# Health Service Delivery Outcomes From Nursing in Genomics: A Scoping Review of the Literature (2012–2025)

**DOI:** 10.1111/inr.70206

**Published:** 2026-07-09

**Authors:** Jordan N. Keels, Joanne Thomas, Kathleen A. Calzone, Laurie Badzek, Sarah Dewell, Emma T. Tonkin, Andrew A. Dwyer

**Affiliations:** ^1^ William F. Connell School of Nursing, Boston College Chestnut Hill Massachusetts USA; ^2^ Genomics Policy Unit Faculty of Life Sciences and Education University of South Wales Pontypridd UK; ^3^ Global Genomics Nursing Alliance (G2NA) and National Institutes of Health, National Cancer Institute, Center for Cancer Research, Genetics Branch Bethesda Maryland USA; ^4^ Global Genomics Nursing Alliance (G2NA) and Ross and Carol Nese College of Nursing Penn State University University Park Pennsylvania USA; ^5^ Global Genomics Nursing Alliance (G2NA) and School of Nursing at Thompson Rivers University Kamloops British Columbia Canada; ^6^ Global Genomics Nursing Alliance (G2NA) and Genomics Policy Unit Faculty of Life Sciences and Education University of South Wales Pontypridd UK; ^7^ Global Genomics Nursing Alliance (G2NA) and William F. Connell School of Nursing, Boston College Chestnut Hill Massachusetts USA

**Keywords:** genomics, midwifery, nursing, nursing education, nursing practice, precision healthcare

## Abstract

**Aim:**

This study aimed to summarize the current state of the science for “health service delivery–oriented outcomes” from nursing in genomics (2012–2025).

**Background:**

Nurses can play a vital role in increasing access to genomic healthcare and improving outcomes for patients, families, and communities.

**Methods:**

We conducted a scoping review of the literature in four databases (2012–2025). Articles were categorized using the Cochrane Collaboration outcome domains and sub‐domains to identify salient topics and synthesize findings.

**Results:**

Of 11,646 retrieved articles, 66 publications reporting “health service delivery–oriented outcomes” were included for analysis. Identified articles spanned three sub‐domains: “service delivery level,” “related to research,” and “societal or governmental.” Within sub‐domains, articles were further categorized into dimensions, the most prominent being “service utilization” under the “service delivery level” sub‐domain. Studies were primarily from anglophone countries and near‐evenly split into interventional and noninterventional studies. Studies reported that nurses working in multidisciplinary and/or interprofessional teams are a cost‐effective means to increase access to genomic healthcare and reduce burden on genetic specialists. Nurses are incorporating genomics in various settings, including oncology, pharmacogenomics, genetic counseling, rare genetic diseases, and symptom science.

**Conclusion:**

Despite evidence of nurses contributing to health system delivery‐oriented outcomes, evidence suggests that nurses are underutilized and underprepared in genomic healthcare.

**Implications for Nursing:**

A significant barrier to integrating genomics into nursing practice is a lack of foundational knowledge and genomic competency across nursing education, clinical practice, and nursing policymakers.

**Implications for Nursing Policy:**

Nursing leadership spanning education, research, practice, and policy domains will be critical for integrating genomics in nursing practice and improving health system delivery‐oriented outcomes.

## 1 Introduction

Scientific advances in genomics have improved disease characterization, guided treatment decision‐making for disease risk management, and informed targeted therapeutic strategies (i.e., personalized or precision healthcare). Precision healthcare includes the integration of genomics into healthcare delivery. Nursing is the largest workforce among all health care providers and is roughly four times greater than the physician workforce (Hassmiller and Wakefield 2022). Integrating genomics into nursing practice is vital to the effective implementation of genomics into routine healthcare. However, there are challenges to widespread adoption, including a shortage of nursing faculty able to teach genomic content as well as systematic oversight, evolving knowledge demands, and leadership recognition to support integration (Tonkin et al. 2025). Globally, 14 countries have invested over $4 billion to overcome barriers to integrating genomics into routine care (Stark et al. 2019). Several initiatives have been launched to establish clinician competency and develop necessary resources and infrastructure to increase provider genomic capacity to streamline and propel implementation into practice (Tonkin et al. 2025).

The initial sequencing of the human genome in 2003 marked the beginning of the “genomic era,” which has now spanned nearly two decades (Collins et al. 2003). Notably, over more than 20 years, there have been repeated calls for nursing to engage in genomic health care (Jenkins et al. 2005; Henly et al. 2011). Nursing leaders and professional organizations have advocated for genomic competencies in nursing education (Conley et al. 2015; Kirk et al. 2011; Lea 2002; Calzone et al. 2018a), building infrastructure to support genomics healthcare (Williams et al. 2016a, 2017, 2016b), and policy advocacy (Kirk et al. 2011; Williams et al. 2018). A 2013 project aimed to establish a “blueprint” for genomic nursing (Calzone et al. 2013a). Part of this work included a systematic literature review, of publications up to May 2012, to assess the evidence on patient outcomes associated with nursing care provided by genomically competent nurses (Calzone et al. 2013a). Only seven of the 415 retrieved articles met the inclusion criteria, precluding a qualitative synthesis.

Recently, an updated systematic literature search was conducted to identify outcomes for nursing/ midwifery involvement in genomics (2012‐2024). Findings were grouped into Cochrane outcome taxonomy domains: (i) consumer‐oriented, (ii) healthcare provider–oriented, and (iii) service delivery–oriented outcomes (Dodd et al. 2018). Two recent publications have reported consumer‐oriented (patient and family) (Keels et al. 2024) and healthcare provider–oriented (clinical and educational) outcomes (Thomas et al. 2023). To date, little is known about health service outcomes and sub‐domains, including (i) service delivery level outcomes; (ii) outcomes related to research; and (iii) societal and governmental outcomes. Importantly, synthesizing evidence on clinical efficacy (i.e., proof‐of‐concept data), feasibility, and integration in healthcare workflow (i.e., service‐level outcomes) is a critical step that could help catalyze the integration of genomics into routine nursing practice. This scoping review reports findings related to service delivery outcomes (2012–2025), summarizes the current landscape of nursing involvement with service delivery outcomes, and highlights recommendations for future directions to advance integration of genomics into nursing and midwifery.

## 2 Methods

We conducted a scoping review of the literature to identify the current state of genomics in nursing/ midwifery and address the broad question “what outcomes are associated with nursing and midwifery practice that incorporates Omics research, principles, technology, and information?” As no human subjects were involved in this scoping review, the project was exempt from ethics board review. The study was registered (PROSPERO 2025 CRD420251063525), and study findings are reported according to the Preferred Reporting Items for Systematic Reviews and Meta‐Analyses extension for the reporting of scoping reviews (PRISMA‐ScR) (Tricco et al. 2018).

In collaboration with a research librarian, we conducted a comprehensive systematic literature search in four databases (PubMed, EMBASE, CINAHL Plus, and Web of Science core collection). The systematic search used medical subject headings (MeSH terms) and keywords (Supplementary Materials). The initial literature search was conducted in July 2022 (publication period: May 2012–July 2022) (Supplementary Materials). The search was repeated to identify articles published in the interim (July 2022–October 2025). Inclusion criteria for eligible studies included: (i) primary research studies published in a peer‐reviewed journal; (ii) studies reporting findings from original studies performed globally (i.e., any country of the world); (iii) studies reporting results/outcomes associated with a nursing activity in omics (i.e., genomics, proteomics, metabolomics, metagenomics, phenomics, and transcriptomics); (iv) studies with an explicit focus on nursing/midwifery activities; (v) published in English; (vi) published since May 2012, i.e., immediately following the publication of the original mixed‐methods systematic literature review (Calzone et al. 2013a). Exclusion criteria included: (i) review articles, letters to the editor, or commentary articles; (ii) reporting secondary or tertiary sources; (iii) studies with no clear nursing/midwifery contribution; (iv) studies with peripheral involvement of nurses/midwives (e.g., part of the study team); (v) studies in which nursing/midwifery activities are not the study focus or without defined outcomes; (vi) not published in English; and (vii) published prior to May 2012.

We used Covidence systematic review software (V2.0, Veritas Health Innovation, Melbourne, Australia, www.covidence.org) to manage the retrieved articles for screening. Identified articles were imported into Covidence and sorted according to the Cochrane Collaboration outcome taxonomy (Dodd et al. 2018). Duplicates were removed automatically. Articles underwent independent dual review. Discrepancies were resolved by a third reviewer. Investigators independently extracted data using a predetermined data collection table. The table was developed specifically for this review to collect title, authors, year, study population, nursing/midwife population, methods, nursing/midwife intervention, genomics focus, summary of study findings/outcomes, and relevant Cochrane outcome domains/sub‐domains.

**FIGURE 1:**
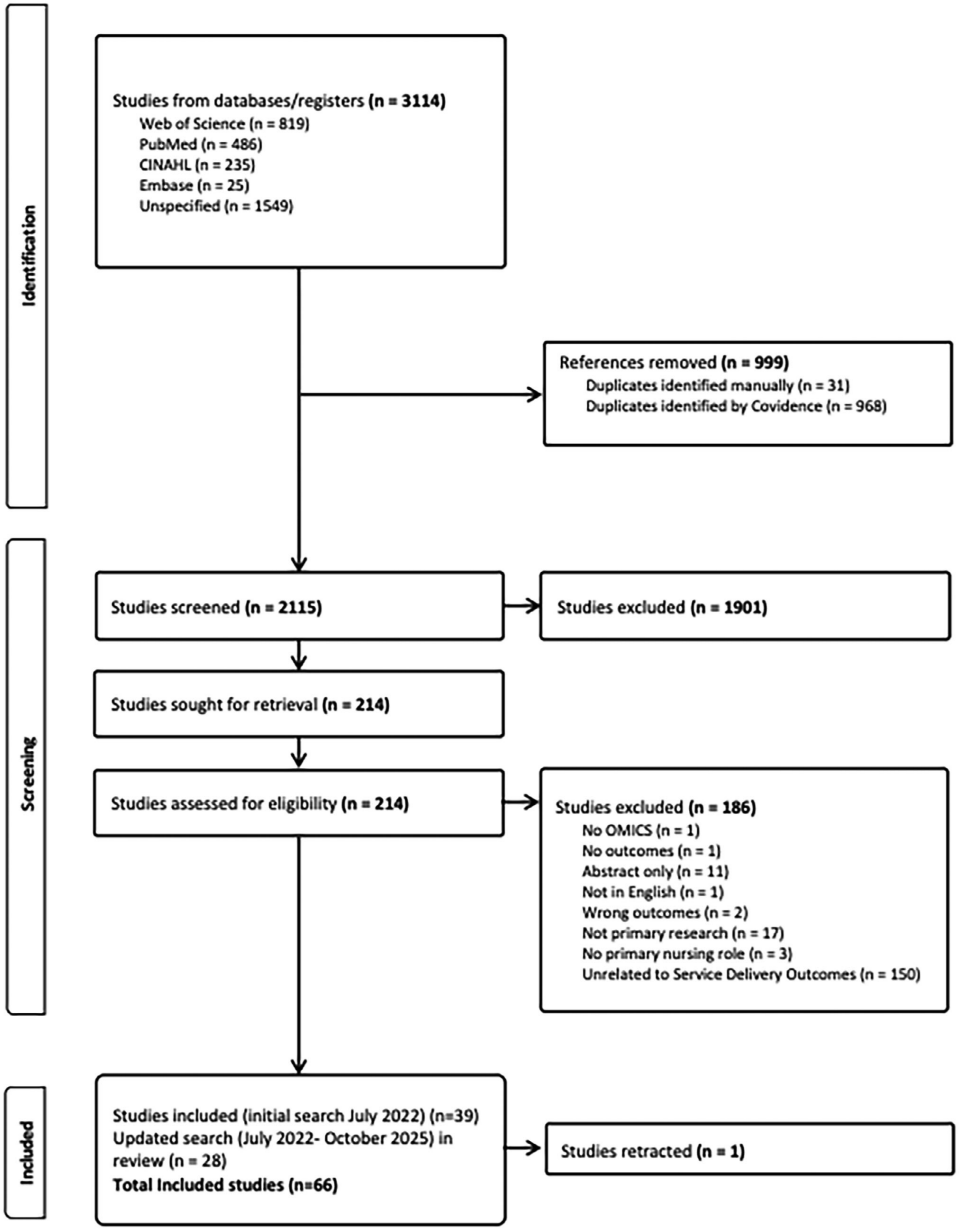
Health Service Delivery Outcomes PRISMA Diagram (July 2022–October 2025).^**^ Prisma diagram from 2012 to 2022 is provided in Supplemental Materials).

Screening identified articles relating to the “health service delivery outcomes” domain of the Cochrane Collaboration taxonomy (Dodd et al. 2018) (Figure 1). Health service delivery outcomes include three sub‐domains: (i) service delivery level (i.e., adverse events, health economic outcomes, and service utilization); (ii) research related (i.e., involvement, recruitment, retention, and feedback from participants); and (iii) societal and governmental outcomes (i.e., health care monitoring, planning, policy, or legislation).

Extracted data were compiled into a master table (Supplementary Materials). Due to methodological variability among studies, we did not assess risk of bias for included studies. Findings are reported narratively using descriptive statistics (i.e., percentages). To synthesize findings on health service delivery outcomes, two investigators reviewed and analyzed identified articles using an iterative process to identify thematic elements (Saunders et al. 2023). After discussing thematic elements with the investigative team, the elements were categorized into sub‐domains for more detailed reporting. Thereafter, thematic analysis was performed to identify salient topics related to nursing in genomics and summarized according to their respective category.

## 3 Results

**FIGURE 2:**
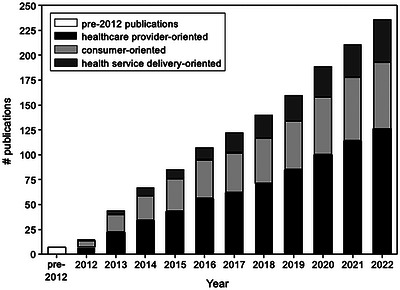
Cumulative genomic nursing publications (2012–2022) by Cochrane outcome domain. The 2012 “blueprint for genomic nursing science” (Calzone et al. 2013a) article only identified 7 articles on nursing and genomics. From 2012 to 2022, there have been 126 articles reporting healthcare provider–oriented (clinical and educational) outcomes, 67 reporting consumer‐oriented (patient and family) outcomes, and 38 reporting health service delivery–oriented outcomes.

The systematic, structured literature search identified 8532 articles. After removing duplicates, 8448 articles underwent title and abstract screening. After initial screening, 615 articles underwent full‐text review, identifying 232 articles for data extraction and analysis. Included studies were classified according to the Cochrane Collaboration outcome taxonomy (Dodd et al. 2018). Three outcomes were identified (i) “healthcare provider–oriented outcomes” 126/232 (54.3%) (Thomas et al. 2023), (ii) “consumer oriented outcomes” 67/232 (28.9%) (Keels et al. 2024), and (iii) “health service delivery oriented outcomes” 39/232 (16.8%) (Figure 2). Study authors reviewed identified publications for retracted articles. One retraction was identified and removed, leaving 38 articles from the initial search (July 2022). Follow‐up searches were conducted up to October 2025, identifying 3114 articles. After duplicates were removed and title/abstract screening, 214 articles underwent full‐text review. A total of 28 articles related to health service delivery–oriented outcomes were selected for inclusion.

**FIGURE 3:**
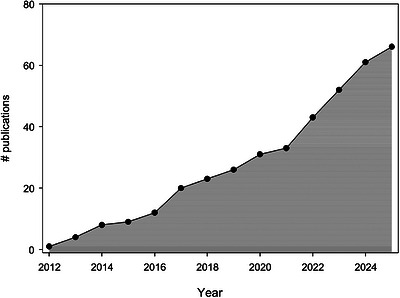
Cumulative publications (2012–2025) reporting health service delivery–oriented outcomes (*n* = 66). Sixty‐six publications reported on health service delivery–oriented outcomes (2012–2025). The number of articles ranged from 1 to 10 per year (median = 3.5, mean 4.7 ± 3.0).

A summary table with study characteristics and key findings for the 66 articles reporting on the “health service delivery outcomes” domain is provided in Supplementary Material. Included articles spanned 14 countries. Three quarters (50/66, 75.7%) were publications from anglophone countries (Bayley et al. 2025; Cruz et al. 2025; Garcia 2025; Jacobs‐McFarlane et al. 2025; Calzone et al. 2018a; Campbell et al. 2017; Braid et al. 2017; Pierce et al. 2017; Knisely et al. 2017; Regan et al. 2019; Butts et al. 2022; Mahon 2013; Shepherd et al. 2014; Mears et al. 2014; Kirk et al. 2014a; McAllister and Schmitt 2015; Percival et al. 2016; Cohen and Nixon 2016; Palomaki et al. 2017; Nisselle et al. 2019; Yoes and Thomas 2020; Wilkinson et al. 2020; Scott et al. 2020; Dunk and Madge 2021; Ingoe et al. 2021; Thompson et al. 2022; Barnhardt et al. 2023; Bendor‐Samuel et al. 2023; Best et al. 2023; Chaigneau et al. 2024; Chiu et al. 2024; Dodson and Layman 2022; Foroughi et al. 2023; Hobbs et al. 2024; Jacobson et al. 2024; Jenkins et al. 2024; Johnson et al. 2024; Kavalieratos et al. 2023; Kehl et al. 2024; Landau et al. 2022; Loughrey et al. 2024; Rives et al. 2023; Shevach et al. 2023; Ward et al. 2022; Calzone et al. 2013a; Smith et al. 2017; Kuhl et al. 2017; Symonds et al. 2018; Temkin et al. 2022; Clark 2016). Three studies were from China (Zayts and Schnurr 2014; Chih et al. 2023; Xia et al. 2022), and one study from Denmark (Byrjalsen et al. 2020), Ghana (Amoaku et al. 2025), India (Singh 2024), Italy (Mordenti et al. 2023), Japan (Goda et al. 2019), Malaysia (Mustaffa et al. 2022), and the Netherlands (Wolters et al. 2022). Six studies included groups from multiple countries (Skirton et al. 2013; Paneque et al. 2017, Heliö et al. 2020; LaRonde et al. 2022; Cordier et al. 2012; Calzone et al. 2018b). Articles reporting service delivery outcomes ranged from 1 to 10 per year (median = 3, Mean 3.2 ± 0.9), and there was a marked uptick (mean 9.3/year) from 2022 to 2024 (Figure 3).

Articles were sorted into three sub‐domains: “service delivery level,” “related to research,” and “societal or governmental.” Of the total included articles, 51/66 (77.2%) were classified in the “service delivery level” sub‐domain (Amoaku et al. 2025; Bayley et al. 2025; Cruz et al. 2025; Garcia 2025; Jacobs‐McFarlane et al. 2025; Mustaffa et al. 2022; Mordenti et al. 2023; Skirton et al. 2013; Mahon 2013; Shepherd et al. 2014; Mears et al. 2014; Zayts and Schnurr 2014; Kirk et al. 2014a; McAllister and Schmitt 2015; Percival et al. 2016; Cohen and Nixon 2016; Palomaki et al. 2017; Paneque et al. 2017; Calzone et al. 2018a; Nisselle et al. 2019; Goda et al. 2019; Heliö et al. 2020; Thompson et al. 2022; Yoes and Thomas 2020; Wilkinson et al. 2020; Scott et al. 2020; Dunk and Madge 2021; Ingoe et al. 2021; Wolters et al. 2022; Barnhardt et al. 2023; Bendor‐Samuel et al. 2023; Best et al. 2023; Chaigneau et al. 2024; Chih et al. 2023; Dodson and Layman 2022; Foroughi et al. 2023; Hobbs et al. 2024; Jacobson et al. 2024; Jenkins et al. 2024; Johnson et al. 2024; Kavalieratos et al. 2023; Kehl et al. 2024; Landau et al. 2022; LaRonde et al. 2022; Loughrey et al. 2024; Rives et al. 2023; Shevach et al. 2023; Singh 2024; Ward et al. 2022; Xia et al. 2022; Clark 2016). Of the remaining articles, 7/66 (10.6%) were in the “related to research” sub‐domain (Campbell et al. 2017; Braid et al. 2017; Pierce et al. 2017; Knisely et al. 2017; Regan et al. 2019; Byrjalsen et al. 2020; Butts et al. 2022) and 8/66 (12.1%) reported on “societal or governmental” aspects (Cordier et al. 2012; Calzone et al. 2013a; Smith et al. 2017; Kuhl et al. 2017; Calzone et al. 2018b; Symonds et al. 2018; Temkin et al. 2022; Chiu et al. 2024). In respect to methodology, 27/66 (40.9%) articles employed quantitative methods (Cruz et al. 2025; Jacobs‐McFarlane et al. 2025; Braid et al. 2017; Pierce et al. 2017; Knisely et al. 2017; Mustaffa et al. 2022; Mordenti et al. 2023; Palomaki et al. 2017; Calzone et al. 2018a; Nisselle et al. 2019; Heliö et al. 2020; Wilkinson et al. 2020; Scott et al. 2020; Ingoe et al. 2021; Thompson et al. 2022; Best et al. 2023; Chih et al. 2023; Hobbs et al. 2024; Jacobson et al. 2024; Jenkins et al. 2024; LaRonde et al. 2022; Rives et al. 2023; Shevach et al. 2023; Xia et al. 2022; Smith et al. 2017; Kuhl et al. 2017; Symonds et al. 2018). Fourteen articles (14/66, 21.2%) used a descriptive approach (Garcia 2025; Butts et al. 2022; Skirton et al. 2013; Shepherd et al. 2014; Percival et al. 2016; Cohen and Nixon 2016; Yoes and Thomas 2020; Chaigneau et al. 2024; Kavalieratos et al. 2023; Kehl et al. 2024; Landau et al. 2022; Loughrey et al. 2024; Cordier et al. 2012; Calzone et al. 2018b). Nine (9/66, 13.6%) reported qualitative research findings (Amoaku et al. 2025; Bayley et al. 2025; Campbell et al. 2017; Mears et al. 2014; Zayts and Schnurr 2014; Kirk et al. 2014a; Paneque et al. 2017; Goda et al. 2019; Chiu et al. 2024) and 5/66 (7.5%) employed mixed methods (i.e., quantitative and qualitative) (Regan et al. 2019; Byrjalsen et al. 2020; Dodson and Layman 2022; Foroughi et al. 2023; Singh 2024). Eleven articles (11/66, 16.6%) were classified as “other” that included quality improvement (*n* = 6) (McAllister and Schmitt 2015; Dunk and Madge 2021; Barnhardt et al. 2023; Johnson et al. 2024; Temkin et al. 2022; Clark 2016), care pathway development (*n* = 2) (Wolters et al. 2022; Ward et al. 2022), methodological advancement (*n* = 1) (Bendor‐Samuel et al. 2023), time study (*n* = 1) (Mahon 2013), and the nursing genomics blueprint (Calzone et al. 2013a). Roughly half of articles reported findings from interventional studies (30/66, 45.4%) (Campbell et al. 2017; Pierce et al. 2017; Mordenti et al. 2023; Mears et al. 2014; McAllister and Schmitt 2015; Cohen and Nixon 2016; Palomaki et al. 2017; Calzone et al. 2018a; Nisselle et al. 2019; Yoes and Thomas 2020; Wilkinson et al. 2020; Dunk and Madge 2021; Ingoe et al. 2021; Barnhardt et al. 2023; Bendor‐Samuel et al. 2023; Chih et al. 2023; Hobbs et al. 2024; Jacobson et al. 2024; Jenkins et al. 2024; Johnson et al. 2024; Kavalieratos et al. 2023; Kehl et al. 2024; Landau et al. 2022; Rives et al. 2023; Shevach et al. 2023; Singh 2024; Xia et al. 2022; Kuhl et al. 2017; Symonds et al. 2018; Clark 2016). A similar number of articles reported results of noninterventional studies (36/66, 54.5%) (Amoaku et al. 2025; Bayley et al. 2025; Cruz et al. 2025; Garcia 2025; Jacobs‐McFarlane et al. 2025; Braid et al. 2017; Knisely et al. 2017; Regan et al. 2019; Byrjalsen et al. 2020; Butts et al. 2022; Mustaffa et al. 2022; Skirton et al. 2013; Mahon 2013; Shepherd et al. 2014; Zayts and Schnurr 2014; Kirk et al. 2014a; Percival et al. 2016; Paneque et al. 2017; Goda et al. 2019; Heliö et al. 2020; Scott et al. 2020; Wolters et al. 2022; Thompson et al. 2022; Best et al. 2023; Chaigneau et al. 2024; Chiu et al. 2024; Dodson and Layman 2022; Foroughi et al. 2023; LaRonde et al. 2022; Loughrey et al. 2024; Ward et al. 2022; Cordier et al. 2012; Calzone et al. 2013a; Smith et al. 2017; Calzone et al. 2018b; Temkin et al. 2022).

### 3.1 Sub‐Domain 1: “Service Delivery Level”

Of the 51 articles reporting on the “service delivery level” subdomain, 21/51 (41%) employed a quantitative methodology (Cruz et al. 2025; Jacobs‐McFarlane et al. 2025; Mustaffa et al. 2022; Mordenti et al. 2023; Palomaki et al. 2017; Calzone et al. 2018a; Nisselle et al. 2019; Heliö et al. 2020; Wilkinson et al. 2020; Scott et al. 2020; Ingoe et al. 2021; Thompson et al. 2022; Best et al. 2023; Chih et al. 2023; Hobbs et al. 2024; Jacobson et al. 2024; Jenkins et al. 2024; LaRonde et al. 2022; Rives et al. 2023; Shevach et al. 2023; Xia et al. 2022). Eleven articles (11/51, 21.5%) were descriptive (Garcia 2025; Skirton et al. 2013; Shepherd et al. 2014; Percival et al. 2016; Cohen and Nixon 2016; Yoes and Thomas 2020; Chaigneau et al. 2024; Kavalieratos et al. 2023; Kehl et al. 2024; Landau et al. 2022; Loughrey et al. 2024). Seven articles (7/51, 9.8%) used qualitative approaches (Amoaku et al. 2025; Bayley et al. 2025; Mears et al. 2014; Zayts and Schnurr 2014; Kirk et al. 2014a; Paneque et al. 2017; Goda et al. 2019) while 3/51 (5.8%) used mixed methods (Dodson and Layman 2022; Foroughi et al. 2023; Singh 2024). Nine articles (9/51, 17.6%) reported on “other” approaches including quality improvement (*n* = 5) (McAllister and Schmitt 2015; Clark 2016; Dunk and Madge 2021; Barnhardt et al. 2023; Johnson et al. 2024), care pathway development (*n* = 2) (Ward et al. 2022, Wolters et al. 2022), time study (Mahon 2013), and methodological development (Bendor‐Samuel et al. 2023). More than half of the studies 26/51 (50.9%) were interventional (Mordenti et al. 2023; Mears et al. 2014; McAllister and Schmitt 2015; Cohen and Nixon 2016; Clark 2016; Palomaki et al. 2017; Calzone et al. 2018a; Nisselle et al. 2019; Yoes and Thomas 2020; Wilkinson et al. 2020; Dunk and Madge 2021; Ingoe et al. 2021; Barnhardt et al. 2023; Bendor‐Samuel et al. 2023; Chih et al. 2023; Hobbs et al. 2024; Jacobson et al. 2024; Jenkins et al. 2024; Johnson et al. 2024; Kavalieratos et al. 2023; Kehl et al. 2024; Landau et al. 2022; Rives et al. 2023; Shevach et al. 2023; Singh 2024; Xia et al. 2022). Twenty‐five articles (25/51, 49.0%) reported findings from noninterventional studies (Amoaku et al. 2025; Bayley et al. 2025; Cruz et al. 2025; Garcia 2025; Jacobs‐McFarlane et al. 2025; Mustaffa et al. 2022; Skirton et al. 2013; Mahon 2013; Shepherd et al. 2014; Zayts and Schnurr 2014; Kirk et al. 2014a; Percival et al. 2016; Paneque et al. 2017; Goda et al. 2019; Heliö et al. 2020; Scott et al. 2020; Wolters et al. 2022; Thompson et al. 2022; Best et al. 2023; Chaigneau et al. 2024; Dodson and Layman 2022; Foroughi et al. 2023; LaRonde et al. 2022; Loughrey et al. 2024; Ward et al. 2022).

The 51 articles in the “service delivery” sub‐domain were sorted into dimensions (i.e., “service utilization,” “health economic outcomes,” “other”). In total, 43/51 (84.3%) related to “service utilization” (Amoaku et al. 2025; Bayley et al. 2025; Cruz et al. 2025; Garcia 2025; Jacobs‐McFarlane et al. 2025; Shepherd et al. 2014; Mears et al. 2014; Kirk et al. 2014a; McAllister and Schmitt 2015; Percival et al. 2016; Cohen and Nixon 2016; Clark 2016; Palomaki et al. 2017; Paneque et al. 2017; Heliö et al. 2020; Wilkinson et al. 2020; Scott et al. 2020; Dunk and Madge 2021; Ingoe et al. 2021; Wolters et al. 2022; Thompson et al. 2022; Barnhardt et al. 2023; Bendor‐Samuel et al. 2023; Best et al. 2023; Chaigneau et al. 2024; Chih et al. 2023; Dodson and Layman 2022; Foroughi et al. 2023; Hobbs et al. 2024; Jacobson et al. 2024; Jenkins et al. 2024; Johnson et al. 2024; Kavalieratos et al. 2023; Kehl et al. 2024; Landau et al. 2022; LaRonde et al. 2022; Loughrey et al. 2024; Rives et al. 2023; Shevach et al. 2023; Singh 2024; Ward et al. 2022; Xia et al. 2022; Calzone et al. 2018a). Two (2/51, 3.9%) articles reported on “health economic outcomes” (Mustaffa et al. 2022; Mordenti et al. 2023). Six (6/51, 11.7%) were considered “other”—primarily focusing on the nursing role in relation to genomics (Skirton et al. 2013; Mahon 2013; Zayts and Schnurr 2014; Nisselle et al. 2019; Goda et al. 2019; Yoes and Thomas 2020).

Sixteen articles (16/51, 31.3%) focused on oncogenetics (Cruz et al. 2025; McAllister and Schmitt 2015; Cohen and Nixon 2016; Clark 2016; Scott et al. 2020; Kehl et al. 2024; Yoes and Thomas 2020; Thompson et al. 2022; Barnhardt et al. 2023; Foroughi et al. 2023; Hobbs et al. 2024; Jenkins et al. 2024; Johnson et al. 2024; Rives et al. 2023; Shevach et al. 2023; Singh 2024). Six articles (6/51, 11.7%) reported on genetic counseling (Mordenti et al. 2023; Skirton et al. 2013; Mahon 2013; Zayts and Schnurr 2014; Percival et al. 2016; Heliö et al. 2020) and rare diseases (8/51, 15.6%) (i.e., sickle cell disease, cystic fibrosis, familial hypercholesteremia, hemophilia), respectively (Amoaku et al. 2025; Jacobs‐McFarlane et al. 2025; Dunk and Madge 2021; Chaigneau et al. 2024; Kavalieratos et al. 2023; Ward et al. 2022; Wilkinson et al. 2020; Ingoe et al. 2021). Three articles focused on pharmacogenomics (Garcia 2025; Jacobson et al. 2024; Dodson and Layman 2022). Two articles (2/51, 3.9%) focused on public health (Mears et al. 2014; Goda et al. 2019), cardiac genetics (LaRonde et al. 2022; Kirk et al. 2014a), newborn screening (Bendor‐Samuel et al. 2023; Nisselle et al. 2019), prenatal screening (Chih et al. 2023; Palomaki et al. 2017), and genomic competency (Calzone et al. 2018a; Paneque et al. 2017). Additional topics included nursing operations (Mustaffa et al. 2022), monogenic diabetes (Shepherd et al. 2014), care planning (Wolters et al. 2022), carrier screening (Best et al. 2023), microbiomics (Landau et al. 2022), genetic testing (Loughrey et al. 2024), transcriptomics (Xia et al. 2022), and hereditary diseases (Bayley et al. 2025).

### 3.2 “Service Delivery Level” Synthesis

Cumulatively, the 51 “service delivery” articles broadly reported on the role and definition of nursing in genomics (Skirton et al. 2013; Zayts and Schnurr 2014). Studies note improved clinical workflow (McAllister and Schmitt 2015; Rives et al. 2023; Shevach et al. 2023; Hobbs et al. 2024; Loughrey et al. 2024) and expanding access to genomics services (Cruz et al. 2025; Shepherd et al. 2014; McAllister and Schmitt 2015; Ingoe et al. 2021; Bendor‐Samuel et al. 2023; Loughrey et al. 2024; Rives et al. 2023), improved equity and access to care (Ingoe et al. 2021; Wolters et al. 2022; Yoes and Thomas 2020; Ward et al. 2022). Findings indicate that nurses can effectively evaluate care pathways to identify service gaps, provide strategic insights, and develop solutions (Kirk et al. 2014a). Nurses have had roles in assessing client comfort with testing/screening (Palomaki et al. 2017), tool development to identify women at an increased risk for hereditary cancers, and facilitating referral to genetic specialists (Clark 2016). Data indicate that integrating nurses into genomic care is cost‐effective and reduces burden in specialty clinics (Mordenti et al. 2023; Thompson et al. 2022; Ingoe et al. 2021). Several articles reported on the effectiveness of nurses and working in multidisciplinary and interprofessional teams (Johnson et al. 2024; Cohen and Nixon 2016). Despite positive outcomes, significant barriers to genetic testing and implementation of genomics into clinical practice remain (Amoaku et al. 2025; Bayley et al. 2025; Singh 2024; Chaigneau et al. 2024; Foroughi et al. 2023; Best et al. 2023). Importantly, nursing “service delivery” interventions have been shown to increase knowledge and confidence among health care providers (Jenkins et al. 2024). While demand for genomic healthcare has grown, policy has not necessarily kept pace with the changing healthcare landscape (Nisselle et al. 2019), and nursing roles/duties in genomics are not always recognized (Goda et al. 2019). These findings point to a need for genomic competency development and corresponding changes in nursing education and training (Inset Box 1).

**Inset Box 1. Summary of key findings across three “Health Service Delivery” sub‐domains**

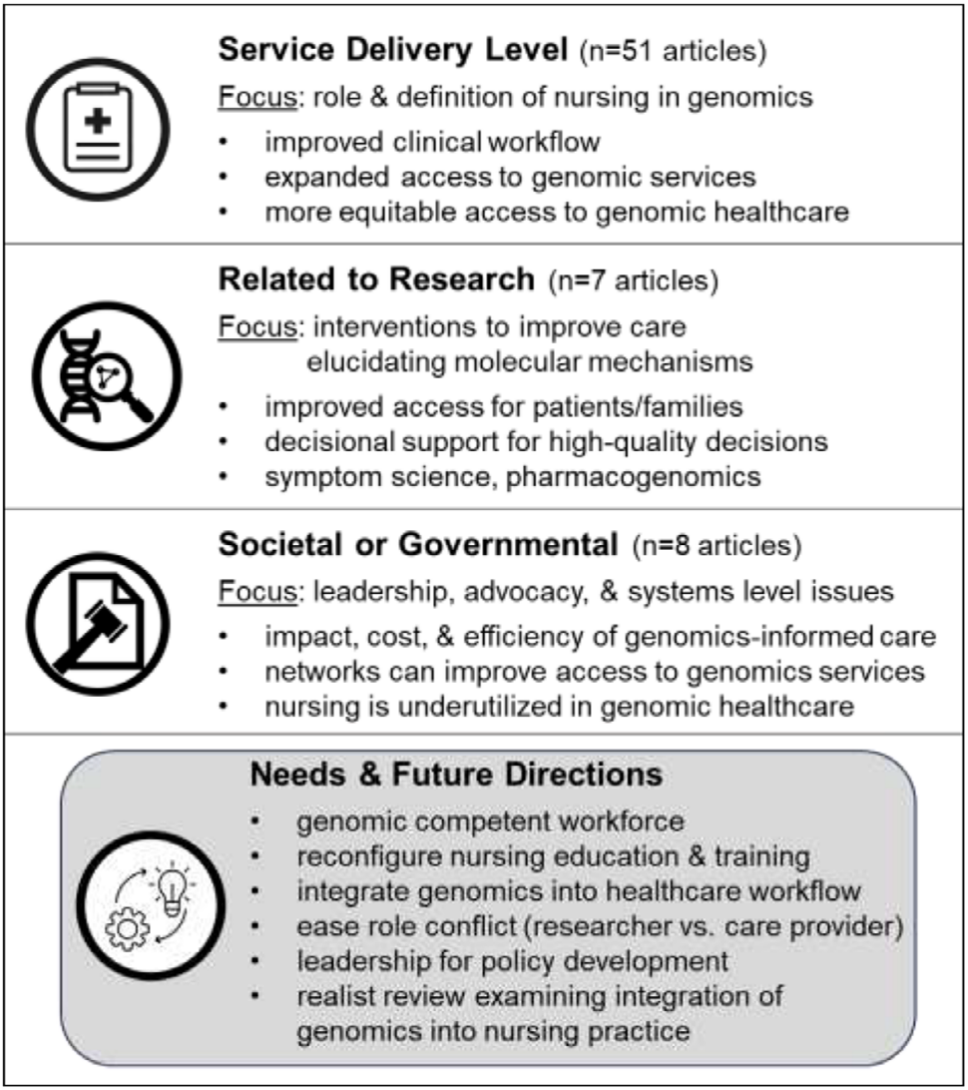

### 3.3 Sub‐Domain 2: “Related to Research”

Seven articles (7/61, 11%) were classified in the “related to research” Cochrane sub‐domain. As for methodology, 3/7 (43%) were quantitative (Knisely et al. 2017; Pierce et al. 2017; Braid et al. 2017). One study employed qualitative methods (Campbell et al. 2017), one was descriptive (Butts et al. 2022), and two articles reported findings from mixed‐methods studies (Byrjalsen et al. 2020; Regan et al. 2019). The majority of articles (5/7, 71%) in the research sub‐domain were noninterventional (Butts et al. 2022, Byrjalsen et al. 2020; Regan et al. 2019; Knisely et al. 2017; Braid et al. 2017), and two (2/7, 29%) were interventional design (Pierce et al. 2017; Campbell et al. 2017). Five articles (5/7, 71%) focused on the “involvement in research” dimension, which included nurse scientist studies or studies with nurses as the population (Regan et al. 2019; Knisely et al. 2017; Pierce et al. 2017; Braid et al. 2017; Byrjalsen et al. 2020). The “recruitment and retention” dimension was the focus of one article (Campbell et al. 2017), and the “other” article reported on adaptation of research during the COVID‐19 pandemic (Butts et al. 2022). Specific areas of focus included genome sequencing (Byrjalsen et al. 2020; Braid et al. 2017), pharmacogenomics (Knisely et al. 2017; Pierce et al. 2017), metabolomics (Butts et al. 2022), informed consent (Campbell et al. 2017), and genomic nursing resource development (Regan et al. 2019).

### 3.4 “Related to Research” Synthesis

Collectively, study findings demonstrate that nurses are engaged in an array of genomic research. Nurse‐led projects aimed to improve patient/family access to care and promote high‐quality decisions (i.e., informed and aligned with values and preferences). Nurses employed person‐centered approaches that considered genomic literacy/numeracy and cultural values to support patient decision‐making (Campbell et al. 2017). Nurses are using sequencing technologies to examine epigenetic influences (i.e., DNA methylation patterns) on health, symptoms, and illness (Braid et al. 2017). Nurse scientists in translational research use next‐generation sequencing technologies to generate “big data” and incorporate a holistic biopsychosocial perspective that considers environment, symptoms, and quality of life to inform more personalized approaches to care (e.g., biobehavioral interventions). Nurses are conducting pharmacogenomic research to improve patient outcomes for traumatic brain injury (Pierce et al. 2017) and identifying drug–gene interactions to reduce adverse events and harm to patients (Knisely et al. 2017). Reports demonstrate nurse's creativity and flexibility to continue ongoing research during the COVID‐19 pandemic (Butts et al. 2022). Study findings also highlight role conflict for nurses as they navigate tension in the dual role as researcher and clinical care provider (Byrjalsen et al. 2020) (Inset Box 1).

### 3.5 Sub‐Domain 3: “Societal or Governmental”

Eight articles (8/61, 13%) related to the “societal or governmental” Cochrane sub‐domain. Regarding methodology, 3/8 (38%) studies were quantitative (Symonds et al. 2018; Kuhl et al. 2017; Smith et al. 2017), two studies (2/8, 25%) were descriptive (Calzone et al. 2018b; Cordier et al. 2012), one used qualitative inquiry (Chiu et al. 2024), and two “other” studies (2/8, 25%). The “other” studies included a quality improvement project (Temkin et al. 2022) and the “blueprint for genomic nursing science” comprising evidence evaluation, advisory panel meetings, obtaining perspectives/feedback from key stakeholders, and a public comment campaign (Calzone et al. 2013a). The majority of studies (6/8, 75%) were noninterventional in design (Calzone et al. 2013a; Cordier et al. 2012; Chiu et al. 2024; Smith et al. 2017; Calzone et al. 2018b; Temkin et al. 2022) and two articles (2/8, 25%) reported results from interventional studies (Symonds et al. 2018; Kuhl et al. 2017). Nearly all articles (7/8, 88%) related to the “health care monitoring” dimension (Kuhl et al. 2017; Smith et al. 2017; Calzone et al. 2013a, 2018b; Cordier et al. 2012; Symonds et al. 2018; Temkin et al. 2022). The remaining article related to the “health care policy” dimension (Chiu et al. 2024). The wide‐ranging topics included oncogenetics (Temkin et al. 2022; Symonds et al. 2018), genomic healthcare legislation (Chiu et al. 2024), genetic counseling (Cordier et al. 2012), genomic nursing blueprint (Calzone et al. 2013a), rare diseases (Smith et al. 2017), newborn screening (Kuhl et al. 2017), and nursing education in genomics (Calzone et al. 2018b).

### 3.6 “Societal or Governmental” Synthesis

Cumulatively, study findings centered on leadership, advocacy, and a call to action for policy development (Chiu et al. 2024). Studies focused on system‐level needs, including accreditation and regulation of genomic nursing roles (Cordier et al. 2012), measuring the impact of genomics‐informed nursing care (Calzone et al. 2013a), and evaluating cost and efficiency (Smith et al. 2017). Importantly, two studies reported on developing a network to improve patient access to genomic healthcare (Kuhl et al. 2017; Temkin et al. 2022). Findings document that nurses are adhering to existing guidelines and are positioned to help implement health system change (Calzone et al. 2018b). Despite existing infrastructure and resources, evidence indicates they are currently being underutilized (Calzone et al. 2018b) (Inset Box 1).

## 4 Discussion

The scoping review identified 66 articles reporting healthcare service delivery–oriented outcomes for nursing/midwifery in genomics (2012–2025. Of the three Cochrane outcome domains, healthcare service delivery had the fewest published articles (Keels et al. 2024; Thomas et al. 2023). The majority of identified articles (50/66, 75.7%) were from high‐income, anglophone countries (i.e., Australia, Canada, Ireland, South Africa, the UK, and the USA). Interestingly, we observed a rather marked uptick in publications between 2020 and 2024. In the first eight years of this review (2012–2019), half of the articles (13/26) were from the USA, with the remaining articles from Australia, China, Japan, and multiple countries (i.e., international collaborations). In contrast, 35 articles were published in the past five years (2020–2024). Forty percent (14/35) were from the USA, while the remainder spanned a more diverse international sampling, including Australia, Canada, Denmark, India, Ireland, Italy, Malaysia, Netherlands, the UK, and multiple countries. Thus, it appears that there is growing interest internationally in assessing and publishing healthcare service delivery outcomes for nursing in genomics.

In total, three‐quarters of the articles (51/66, 77.2%) reported on the “service delivery” subdomain. Findings indicate that nurses and advanced practice registered nurses (i.e., nurse practitioners, midwives, clinical nurse specialists, nurse anesthetists) working in multidisciplinary and/or interprofessional teams are a cost‐effective means to increase access to genomic healthcare and reduce specialist burden (Mordenti et al. 2023; Thompson et al. 2022; Ingoe et al. 2021). Nursing has contributed to advancing person‐centered approaches to genomic healthcare decisional support/decision‐making that considers health literacy/numeracy, culture, and preference (Campbell et al. 2017). Nurse scientists have made significant contributions to symptom science that inform more tailored approaches to care (i.e., precision healthcare) (Knisely et al. 2017; Pierce et al. 2017; Braid et al. 2017). Nurses working in genomics often experience a lack of role clarity (Goda et al. 2019)—a finding that was mirrored in the “research subdomain” where nurses felt a tension between their respective clinical and research roles (Byrjalsen et al. 2020). Together, findings underscore a need for nursing leadership and a call to action and policy advocacy to support nurses in incorporating genomics into practice. Previously, both US and UK (i.e., National Health Service) policy reforms regarding genomic service provision included nursing (Government 2020). However, the US National Institutes of Health National Institute for Nursing Research has recently redirected funding that was previously allocated for nurses in genomics (Kurnat‐Thoma et al. 2021). Moreover, credentialing organizations (i.e., American Association of Colleges of Nursing) have moved away from including genomics in updated essentials for nursing education in the USA (Connors and Schorn 2018). Thus, urgent action is needed to reestablish direction and set a clear course to ensure that the discipline is prepared for, responsive to, and engaged with the growing demand for genomic healthcare and expanding use of genomics in healthcare delivery (Inset Box 1).

Competencies are critical for equipping the nursing workforce with the requisite knowledge, attitudes, and skills to deliver high‐quality, genomics‐informed care. The present data and several systematic reviews highlight that nurses are underutilized in meeting the need for genomics healthcare (Keels et al. 2024; Thomas et al. 2023; Cao et al. 2025; McLaughlin et al. 2024; Gusen et al. 2025; Carpenter‐Clawson et al. 2023). A number of countries have assessed nurses’ genomic knowledge and practice of genomics‐informed care (Thomas et al. 2023), and competency frameworks exist for the USA (Calzone et al. 2024; Calzone and Badzek 2025), the UK (Kirk et al. 2014b), Japan (Murakami et al. 2020), and Europe (Skirton et al. 2010). In addition, a competency framework for precision healthcare has been recently introduced in the USA (Schultz et al. 2025). Importantly, competencies can establish minimum standards for practice (Calzone and Badzek 2025; Dickman et al. 2025). As such, recognized competencies can help clarify nursing roles, delineate boundaries relating to the scope of practice, and identify opportunities for career progression (Keels et al. 2024; Thomas et al. 2023; Cao et al. 2025; McLaughlin et al. 2024; Gusen et al. 2025; Park et al. 2024). The Global Genomics Nursing Alliance (G2NA) (Tonkin et al. 2025) is currently working to develop global, genomic nursing competencies for all nurses regardless of educational preparation, nursing role, or structure of health service delivery (Tonkin et al. 2025). International collaboration and global efforts will be foundational for integrating genomics into nursing practice in settings beyond high‐income, anglophone countries.

Notably, numerous nursing competency frameworks exist—yet the mere existence of a framework does not ensure sustained practice change. Importantly, implementation is blocked when policies that articulate competencies are disconnected from real‐world settings, where nurses face numerous competing clinical demands and structural barriers. Structural barriers for incorporating genomics into nursing include a lack of genomics integration into clinical workflow, such as genomic data being inaccessible at the point of care (e.g., pharmacogenomics in the electronic health record) (El Rouby and Johnson 2025). As a result of these structural barriers, nurses may lack the appropriate time bandwidth to integrate genomics into the nursing workflow (Calzone et al. 2025).

With the push toward competency‐based nursing education (American Association of Colleges of Nursing 2021), a tension has emerged between establishing specialized competencies (e.g., informatics, mental health, palliative care) and finding sufficient space within the curriculum to embed them into practice. As genomics is a lifespan competency (Calzone et al. 2013b), it may be most effective to view genomic nursing as a “lens” through which nurses practice—regardless of patient population, disease context, or care setting (Dewell et al. 2020). Such a parsimonious approach integrates genomics into existing frameworks, mitigating “competency fatigue” and the overproliferation of requirements (Limoges et al. 2024). To achieve this, cross‐sector collaboration among nurse researchers, nursing educators, policymakers, and point‐of‐care clinicians is essential to develop a unified strategic direction for implementing genomics alongside other emerging priorities like artificial intelligence and machine learning (Limoges et al. 2024).

To embed genomic competencies in nursing education and training requires greater capacity for nursing faculty to teach genomic content—yet faculty preparation is highly uneven (Thomas et al. 2023). Unfortunately, the level of genomic expertise of many nursing faculty is similar to the students they teach (Read and Ward 2016; Dante et al. 2025). Similarly, there is a need for clinical instructors and educators (i.e., professors of the practice) to have a solid foundation in genomics to support learners. In tandem, there is a crucial need for nursing students to have clinical experiences delivering genomics‐informed care. Such experiences may come from in vivo encounters or using clinical simulation to reinforce genomic content (White et al. 2025; Dwyer et al. 2025). Beyond nursing education, another complicating factor is that many practicing nurses do not have the requisite knowledge or competencies to practice genomic‐informed care (Keels et al. 2024; Thomas et al. 2023). Thus, developing the active nursing workforce is a parallel need. In summary, a crucial challenge to nursing is that there is often no foundation in genomics to build upon, thus creating a vicious cycle. For example, nursing faculty who lack core genomic knowledge are less effective at integrating genomics into nursing education. Similarly, nurses who completed their education and training prior to the genomic era are ill equipped to incorporate genomic competencies into clinical practice. This implementation gap is further amplified when new graduates, who did not have genomic content in their nursing curricula, enter the nursing workforce.

Breaking this vicious cycle requires an acknowledgment of the broader systemic barriers that pose barriers to implementation. As a female‐dominated profession, gendered nursing roles affect role delineation and interprofessional interactions (Piervisani et al. 2025). Further, there exists an uncomfortable disconnect between nursing education and clinical practice (Greenway et al. 2019; Leonard et al. 2016). Medical educators typically remain active in clinical practice. In contrast, many nurse educators are removed from direct care roles and the rapidly evolving clinical evidence base of our complex healthcare ecosystem. Such disconnection stifles the virtuous, ongoing (so‐called “figure‐eight”) generation and transfer of knowledge and information between the academic and clinical domains. Re‐envisioning academic–practice partnerships is one way to address the uncoupling. Innovative roles such as a nurse implementation scientist (with protected time) could provide point‐of‐care expertise and serve as a translational bridge to support educators in integrating emerging content to meet evolving clinical needs. Such a role would require significant institutional commitment as an additional “cost center” until data could assess return on investment (Abujaber and Nashwan 2025).

Moreover, when nurses in leadership roles lack core genomic knowledge, genomic healthcare is underprioritized. Importantly, both the International Society of Nurses in Genetics (Hickey et al. 2018) and the Global Genomics Nursing Alliance (G2NA) (Tonkin et al. 2025) are collaborating across institutions and internationally to catalyze leadership and propel integration of genomics into nursing practice. It merits noting that leadership support across settings is essential for integrating and strengthening genomics in nursing practice. Leadership support can include investing in training champions to lead education and integration efforts in a specific academic or clinical setting (Jenkins et al. 2015; Jenkins and Calzone 2014); mapping genomic competencies to practice and/or educational standards to facilitate integration (Willey et al. 2025); developing and implementing point‐of‐care decision support in clinical settings (Sharma et al. 2025); developing single concept learning interventions (Prochazkova et al. 2019); and/or implementing interprofessional team education (Ersig et al. 2025). Leadership must also recognize that no health discipline exists in a silo and that genomics is a collaborative “team sport” involving multiple disciplines (Ma et al. 2024). To date, interdisciplinary education at the undergraduate and graduate levels has been piecemeal (Lawlis et al. 2014). However, it is plausible that embedding a collaborative approach early in clinician formation could shape mental models and a culture of multidisciplinary collaboration in healthcare teams—shifts that could have a significant impact on such long‐standing implementation challenges.

Importantly, leaders should acknowledge that ongoing interventions and “boosters” are required until the nursing workforce demonstrates a solid, foundational understanding of genomics. Additionally, practicing nurses and nursing faculty trained in genomics require continuing education as genomics knowledge continues to rapidly expand. Leaders can advocate, develop, and support policies that enable genomic integration and foster research to measure outcomes (Cao et al. 2025). For sustainable change to occur, it is critical that leadership has sufficient training to understand the relevance of genomics to nursing practice and the importance of top‐down support for capacity‐building initiatives. Additionally, it is critical to appreciate rapidly changing genomic technologies and evolving evidence‐based applications to practice. Integrating genomics into healthcare delivery is a dynamic process. Thus, capacity building should be viewed as an ongoing endeavor and not simply a “one and done” task.

Both the literature and professional genomics nursing organizations highlight the importance of and opportunities for genomics‐informed nursing practice. Genomics does not have to be an “add‐on” that increases nursing workload and burden. Rather, genomics is a lens that can be integrated into the nursing process (Dewell et al. 2025; Davis et al. 2025). Evidence demonstrates that nurse‐led interventions can increase access to genomic healthcare and support high‐quality decisions (i.e., informed and aligned with patient values and preferences) (Kronk and Subasic 2025; Park et al. 2024; Jull et al. 2021; Park et al. 2025). As demonstrated in examples from oncology (Cruz et al. 2025; Temkin et al. 2022; Symonds et al. 2018; Dickman et al. 2025) and rare diseases (Singh 2024; Chaigneau et al. 2024; Foroughi et al. 2023; Best et al. 2023; Amoaku et al. 2025; Bayley et al. 2025), nurses have a key role in shaping person‐centered approaches to genomic healthcare across the full continuum of care (Katapodi et al. 2023). Despite exemplars, there is a persistent gap between the importance that nurses place on genomics‐informed care and actual clinical practice (Keels et al. 2024; Thomas et al. 2023; Carpenter‐Clawson et al. 2023; Gusen et al. 2025). Notably, a recent study highlighted that nurses’ knowledge of genomics has changed little over the past decade (Calzone et al. 2025), thus hindering genomics‐informed nursing care. Implementation science has long been proposed as a pathway to integrate genomics into nursing practice and system‐level change (Williams et al. 2017). Moreover, a number of initiatives have helped create a framework for successfully integrating a new genomics competency (Jenkins et al. 2015) and delineating benchmarks for tracking progress in the integration of genomics in nursing practice (Tonkin et al. 2020).

Findings from the present work and two prior scoping reviews examining healthcare provider– and consumer‐oriented outcomes (Keels et al. 2024; Thomas et al. 2023) echo other recent systematic literature reviews examining implementation of genomics by primary care providers (Dunlop et al. 2025; Walters et al. 2024), allied health professions (Anandam et al. 2026), and pharmacists (Coumau et al. 2025). Building upon the landscape presented in the present work, future research should generate rigorously appraised evidence that is required to inform policy and potentially spur system redesign. Specifically, future systematic reviews should focus on specific populations or care settings (e.g., oncology and rare diseases) to enhance the actionability of findings. Similarly, realist reviews informed by implementation science and focusing on context, mechanisms, and outcomes could help explain the “how and why” genomic interventions work in specific nursing contexts. We must provide evidence‐based, proof‐of‐concept data that clearly articulate genomic outcomes with nursing‐sensitive metrics.

Despite abundant opportunities for nurses to help realize the full potential of genomics to improve outcomes for patients, families, communities, and health systems, integration has been lacking. This scoping review found that of the three Cochrane outcome domains, service delivery outcomes were the least reported. Yet, there has been an increase in the volume of papers over the past four years focusing on implementation and service redesign in nursing. It is plausible that the observed trend may be the result of critical drivers of practice change, including policy changes; growing evidence base for genomics to improve outcomes; shifting consumer expectations surrounding genomic and precision healthcare; and/or infrastructure and service redesign facilitating integration of genomics into routine clinical care (e.g., mainstreaming). Further monitoring is warranted to track the integration, growth, and sustainability of genomics into nursing education, practice, and research.

Relative strengths of this scoping review include the structured approach employed to retrieve relevant articles, the rigorous dual review process, the use of the Cochrane outcome taxonomy, and the updated literature search, ensuring a comprehensive and timely synthesis. There are a number of limitations that are worth noting. First, it is possible that we may have inadvertently excluded some relevant articles. For example, nurses were a part of a multidisciplinary or interprofessional team in many publications. As such, it is possible that we could not identify nursing authorship or contribution. As the methods of included articles were quite variable and heterogeneous, we did not conduct a formal assessment of bias. Lastly, the majority of articles were from anglophone, high‐income countries, so caution is merited in attempting to extrapolate findings to other healthcare settings (i.e., low‐middle income countries).

## 5 Conclusions

This scoping review provides a current state of the science on nursing‐related genomic healthcare service delivery outcomes. Ample evidence underscores the promise of genomics for improving outcomes for patients, families, communities, and health systems. Nurses integrating genomics into clinical practice can help increase access to person‐centered genomic healthcare and may reduce precision healthcare inequities. It takes roughly 17 years on average for research discoveries to be incorporated into clinical practice (Morris et al. 2011). More than 20 years into the “genomic era,” data suggest that nurses are not fully prepared to deliver genomics‐informed care. Accordingly, nurses have been underutilized, and we have yet to deliver the full promise of genomic healthcare for improving health outcomes for patients, families, and communities. Current and future work must shift from merely documenting implementation gaps to generating rigorously appraised, proof‐of‐concept data incorporating nursing‐sensitive metrics to clearly articulate the return on investment and making a clear business case for genomics‐informed nursing practice, driving health service delivery outcomes. Such efforts will distinguish the future from the past patterns of the previous two decades.

### 5.1 Implications for Nursing and Health Policy

The challenges are multifactorial, spanning nursing education, accrediting organizations, the clinical workforce, and nursing leadership. To enhance healthcare service delivery outcomes, multilevel efforts are needed that may include embedding genomic competencies into nursing education/training; developing genomic capacity of nursing faculty; having accreditation standards that include genomics and precision health, clearly defining roles and scope of practice for the genomic workforce; using validated measures to assess outcomes; applying implementation science to spur health system‐level change; and strong advocacy to shape policy that funds infrastructure to build a nursing workforce capable of delivering genomics‐informed care.

## Author Contributions

JNK: Formal analysis, investigation, data curation, writing – original draft, writing – review and editing. JT: Formal analysis, investigation, data curation, writing – review and editing. KAC: Conceptualization, validation, investigation, project administration, resources, writing – review and editing. LB: Conceptualization, writing – review and editing. SD: Conceptualization, writing – review and editing. ETT: Conceptualization, methodology, formal analysis, investigation, validation, writing – review and editing. AAD: Conceptualization, validation, formal analysis, investigation, project administration, resources, supervision, validation, visualization, writing – original draft, writing – review and editing.

## Conflicts of Interest

The authors declare no conflict of interest.

## Funding Information

Dr. Dwyer received funding support from the Josiah Macy Jr. Foundation (MFS‐23‐02) and National Institutes of Health Eunice Kennedy Shriver National Institute of Child Health and Human Development (1P50HD104224‐01, “Massachusetts General Hospital Harvard Center for Reproductive Medicine”) for this work. JT Thomas received doctoral study funding from the Knowledge Economy Skills (KESS2) Scholarship and the Genomics Partnership Wales. The funders had no role in the design of the study; in the collection, analyses, or interpretation of data; in the writing of the manuscript; or in the decision to publish the results.

## Supporting information




**Supporting File 1**: inr70206‐sup‐0001‐Figure S1.docx

**Supporting File 2**: inr70206‐sup‐0002‐Table S1.xlsx
