# Supplementary material for: Health Service Delivery Outcomes From Nursing in Genomics: A Scoping Review of the Literature (2012–2025)
Source: Int Nurs Rev. 2026 Jul 9;73(3):e70206. doi: 10.1111/inr.70206 (PMC13351011; doi:10.1111/inr.70206)
Supplement: Supplementary file 1 — Supporting File 1: inr70206‐sup‐0001‐Figure S1.docx [file INR-73-0-s001.docx]

PRISMA flow diagram for the systematic review (2012-2022)

Records identified from search

(n = 8,532)

**Identification**

Duplicate records removed

(n = 84)

Records excluded

(n = 7,833)

Records screened

(n = 8,448)

Reports excluded (n = 383)

- abstract/poster only (n=227)
- not primary research (n=60)
- no nursing/midwife contribution (n=43)
- not Omics (n=31)
- unable to retrieve full text (n=10)
- outcome(s) not reported (n=8)
- duplicate (n=3)
- not published in English (n=1)

Duplicate (n = 3)

Abstract only (n = 227)

Literature review (n = 2)

Not primary research (n = 58)

Outcome(s) not reported (n = 8)

Unable to locate full text (n = 10)

Article not published in English (n = 1)

No specific OMICS activity/intervention (n = 31)

No nursing or midwifery contribution in study (n = 43)

Studies assessed for eligibility

(n = 615)

**Screening**

Studies included in review (n=232)

- Healthcare provider oriented outcomes (n=126)
- Consumer oriented outcomes (n=67)
- Health service delivery outcomes (n=38)

Health service delivery records retracted

(n = 1)

**Included**
